# Dysbiosis in Head and Neck Cancer: Determining Optimal Sampling Site for Oral Microbiome Collection

**DOI:** 10.3390/pathogens11121550

**Published:** 2022-12-16

**Authors:** Dheeraj Pandey, Michal Szczesniak, Julia Maclean, Howard Chi Ho Yim, Fan Zhang, Peter Graham, Emad M. El-Omar, Peter Wu

**Affiliations:** 1 St George and Sutherland Clinical Campuses, School of Clinical Medicine, University of New South Wales, Sydney 2052, Australia; 2 Department of Gastroenterology and Hepatology, St George Hospital, Sydney 2217, Australia; 3 Department of Speech Pathology, St George Hospital, Sydney 2217, Australia; 4 UNSW Microbiome Research Centre, UNSW, St George Hospital, Sydney 2217, Australia; 5 Department of Radiation Oncology, St George Hospital, Sydney 2217, Australia

**Keywords:** oral microbiome, dysbiosis, head and neck cancer, salivary microbiome, chemoradiation therapy

## Abstract

Recent research suggests that dysbiosis of the oral microbial community is associated with head and neck cancer (HNC). It remains unclear whether this dysbiosis causes chemo-radiotherapy (CRT)-related complications. However, to address this question, it is essential to determine the most representative oral site for microbiome sampling. In this study, our purpose was to determine the optimal site for oral sample collection and whether the presence of HNC is associated with altered oral microbiome from this site. In 21 newly diagnosed HNC patients and 27 healthy controls, microbiome samples were collected from saliva, swabs from buccal mucosa, tongue, hard palate, faucial pillars and all mucosal sites combined. Microbial DNA was extracted and underwent 16S rRNA amplicon gene sequencing. In healthy controls, analysis of observed taxonomic units detected differences in alpha- and beta-diversity between sampling sites. Saliva was found to have the highest intra-community microbial diversity and lowest within-subject (temporal) and between-subject variance. Feature intersection showed that most species were shared between all sites, with saliva demonstrating the most unique species as well as highest overlap with other sites. In HNC patients, saliva was found to have the highest diversity but differences between sites were not statistically significant. Across all sites, HNC patients had lower alpha diversity than healthy controls. Beta-diversity analysis showed HNC patients’ microbiome to be compositionally distinct from healthy controls. This pattern was confirmed when the salivary microbiome was considered alone. HNC patients exhibited reduced diversity of the oral microbiome. Salivary samples demonstrate temporal stability, have the richest diversity and are sufficient to detect perturbation due to presence of HNC. Hence, they can be used as representative oral samples for microbiome studies in HNC patients.

## 1. Introduction

The incidence of head and neck cancer (HNC) and associated mortality have been on the rise in Australia and around the world [1,2]. Head and neck cancers are heterogenous in terms of their structural and molecular origin making it challenging to develop targeted therapeutics. Conventional therapies, specifically chemotherapy and radiation treatment (CRT) have improved vastly over the last two decades in terms of cure rate and mitigating collateral organ damage, but they still leave survivors with sometimes permanent side-effects, highlighting the need for treatment stratification using validated biomarkers to improve treatment outcomes and reduce toxicity [3].

Emerging evidence suggests oropharyngeal microbiota may play a role in HNC carcinogenesis as well as in toxicities induced by CRT. While studies have shown that oropharyngeal dysbiosis is present in those who were diagnosed with HNC when compared with non-cancer controls, any causative link between dysbiosis and cancer remains unproven [4,5,6,7,8]. Also, there are studies linking chemoradiation therapy to changes in microbial community structure [9,10] and data exist implicating dysbiosis as a risk factor for severe oral mucositis [11,12,13]. The clinical relevance of these findings in treatment outcomes and complications remains unclear. In particular, the critical question regarding the potential role of the microbiome in the genesis of late toxicity (fibrogenesis, neuromuscular damage) following CRT has never been addressed. These limited data, together with strong biological plausibility, support the notion that the pathogenic link between the microbiome and these phenomena warrants further systematic investigation.

At a very fundamental level, however, there are several methodological challenges that first need to be resolved. The oral cavity harbours microbial community with extensive diversity including at least 15 identified phyla spread out in distinct identifiable clusters of varying composition across different niches [14,15]. Due to unique prevailing physical and biological conditions these sites offer varied habitats contributing to varied composition and diversity of microbiota. Microbiome profiles are shown to differ across at least eight different target sites for microbiota collection in the oral cavity and oropharynx (saliva, buccal mucosa, hard palate, tonsils, tongue, faucial pillars, keratinised gingiva, and gingival plaques), as well as the collection method used [16,17,18,19]. Furthermore, a range of host genetic and lifestyle factors very likely impact the inter-subject variability of the oral microbiota composition [20], and the extent of this variability remains unknown.

While distinct microbiome profiles at different oral sites give an opportunity to study site specific changes in oral microbiomes for individual and local diseases [21,22], it also creates a hurdle when studying systemic diseases such as cancer where identifying overall changes in oral microbiome composition is important [22]. Not only is collecting samples from different sites for analysis logistically difficult and expensive, but it can also be problematic when comparing results from one study to another due to the selection of different sites and different collection methods utilised [23]. Additionally, temporal changes of oral microbiome due to daily oral hygiene treatment, varied dietary intake and exposure to risk factors such as smoking and drinking further complicate comparison of results from one study to another [15]. Furthermore, there is very little evidence regarding the stability of intra-subject oropharyngeal microbiome over time [24]. Thus, it is important to select a site and develop a method of collection that is easily replicable, minimally intrusive, and not time intensive, while sufficiently representing inherent diversity from all oral and oro-pharyngeal sites.

Currently, there is limited consensus on the optimal microbiota sampling site. Previous studies on oral microbiome profile are in broad agreement that plaque is distinctive from other oral sites and that saliva constitutes a diverse microbiota that has been shown to be a conglomerate of bacteria derived primarily from mucosal surfaces of the cheeks, tongue, throat, and the tonsils [20,25]. However, studies have also found that mucosal sites like buccal mucosa and tongue have higher or similar alpha diversity and close resemblance to microbial profile of saliva [17,26]. These results are promising; however, there are factors that limit their generalisation, such as use of non-standardised protocols, small sample sizes, cross-sectional study designs, and samples from single time points.

Thus, in the present study we aimed to determine the optimal site for sampling oral microbiomes by evaluating diversity, stability and overlap of oral microbiomes collected from saliva and distinct mucosal sites in the oropharyngeal region. These findings will highlight site-specific differences in oral microbiome composition and provide indications of whether microbiomes from saliva can be used for comparative studies involving diseases such as HNC.

## 2. Materials and Methods

### 2.1. Study Design

An observational study in two cohorts: (1) healthy controls and (2) newly diagnosed HNC patients. Additional test-retest component in healthy controls to assess temporal within-subject variance. Each participant underwent screening and enrolment where written informed consent was obtained; this was followed by collection of demographic data and microbiome sample collection. Healthy controls were resampled 4–6 weeks later.

### 2.2. Participants

A total of 27 healthy controls aged 42 to 90 years (mean 63.6 ±  s.d. 12.4 years) were recruited from the suburbs of Georges River, Canterbury, and Bankstown and Sutherland shire, which contribute a majority of patients admitted at St George Hospital, and 21 pre-treatment head and neck cancer patients aged 42 to 83 years (mean 56.9 ±  s.d. 14.6 years) were recruited at the St George Hospital Cancer Care Centre. Both healthy controls and HNC subjects were enrolled in the study between Feb 2019 and June 2020. Inclusion criteria for healthy controls: adult participants who do not have HNC (mucosal squamous carcinoma of the tongue, buccal mucosa, tonsil, palate, hypopharynx, larynx). Inclusion criteria for HNC patients: adult patients who have newly diagnosed HNC (mucosal squamous carcinoma of the tongue, buccal mucosa, tonsil, palate, hypopharynx, larynx) about to undergo curative chemoradiation. Exclusion criteria for both groups: unable to provide consent; age < 40 years; other pre-existing disorder known to cause pharyngeal dysphagia (eosinophilic oesophagitis, achalasia, oesophageal cancer, any neuropathic/myopathic disorders known to cause pharyngeal dysphagia, e.g., MVA, MND, Parkinson’s, inflammatory myopathy); current/intended pregnancy; recent antibiotic exposure (3 months); and other co-morbid conditions that preclude inclusion in the study.

### 2.3. Sample Collection

Sample collections for healthy controls and HNC patients were conducted either at St George Hospital or at the participants’ homes. Non-HNC healthy subjects were sampled at two different time-points to assess temporal changes in microbiomes. HNC patients were sampled soon after diagnosis but before CRT treatment commenced. Participants were instructed not to eat or drink for at least 2 h prior to collection. The specimens included saliva, four mucosal tissues (tongue, buccal mucosa, faucial pillars, hard palate), and a single cumulative swab of all four mucosal sites. For saliva, subjects were asked to let saliva collect in the mouth for 10–15 s or until a sufficient amount was collected to spit out. They were asked to expel saliva into a sterile jar, which was then transferred into a 2 mL eNAT tube with DNA stabilising buffer. Mucosal sites were sampled using sterile rayon swabs (Floqswab). Four mucosal sites were sampled individually, and a cumulative mucosal specimen (all 4 mucosal sites) was taken by sampling all 4 sites in a single sweep. Swabs were then stored in 2 mL eNAT tubes with DNA stabilising buffer. In addition, 4 negative controls—2 of which were collected by exposing swabs to air for 10 s and stored in 2 mL eNAT tubes with DNA stabilising buffer to mimic mucosal sample collection; another 2 were collected by transferring 2 mL eNAT buffer to a sterile jar and pipetting it back to a eNAT tube in order to mimic collection of saliva. All samples were aliquoted and stored at −80 °C until use.

### 2.4. DNA Extraction

Total genomic DNA was extracted from oral samples using QIAamp DNA Mini Kit as per manufacturer’s instructions. Oral samples were thawed on ice, 30 μL of proteinase k (20 mg/mL) and 600 μL of Buffer AL were added to 600 μL of the sample. The samples were incubated at 56 °C for 1 h. After incubation, cells were mechanically lysed using the Qiagen Tissue-Lyser II (Qiagen, Germantown, MD, USA #85300) at 30 Hz for 2 min. DNA was purified and eluted using nuclease-free water. Extracted DNA quantity was measured using Qubit Fluorometric Quantification. Presence of bacterial DNA was determined by PCR amplification of V3 and V4 regions of rRNA 16S bacterial gene using forward primer 16S-341F and reverse primer 16S-805R. The resulting product was visualised using agarose gel electrophoresis.

### 2.5. Sequencing, Library Preparation and Analysis

Samples containing DNA were sequenced for library preparation using Illumina Miseq Pair-end sequencing at the Ramaciotti Centre in University of New South Wales. Amplicon data were quality controlled with dada2 [27] embedded in qiime2 [28]. Host contamination was removed using Bowtie 2 (version 2.4.2) [29]. Taxonomy annotation of the data was performed using a qiime2 feature classifier plugin with the relevant greengene database and ITS database, respectively. R (version 4.0.4) packages qiime2R [30] and phyloseq [31] were employed for diversity analysis.

### 2.6. Statistical Analysis

Baseline demographics were compared with Yates’s chi-squared test for categorical variables and unpaired t-test for continuous measures.

A Mann–Whitney–Wilcoxon test was applied in the comparison of the means of the Alpha diversities between different groups. To access the significance of disease and other metadata variable effects between two distance matrices in the Beta diversity analysis, Adonis (permutational multivariate analysis of variance using distance matrices) was used to permute the distance matrix 999 times to yield *p*-values and ESS (explained sum of squares).

Sample size estimation was performed using shinyMP web application [32] using control datasets pre-set on Human Microbiome Project (HMP) protocols (including saliva). Briefly, by using HMP saliva sample data as ‘Control group’ and data from the highly prevalent and less represented bacterial genera in a preliminary set of our samples (including *Streptococcus, Candidatus, Cutibacterium, Gemella, Pseudomonas, Actinomyces, Pseudopropionibacterium, Aggregatibacter, Corynebacterium, Staphylococcus, Veillonella, Parvimonas, and Micrococcus*) as ‘Case group’, we compared the statistical power of different sample sizes ranging from 5 to 100 samples, using the Wilcoxon-Mann-Whitney test. For a number of 20 samples in both groups the resulting power was 0.96.

## 3. Results

### 3.1. Study Population and Demographics

A total of 48 subjects were recruited in the study, 21 with recently diagnosed HNC and 27 non-HNC controls. Population demographics are shown in Table 1. The two study cohorts were comparable in terms of mean age and gender with 63.6 years and 70.3% male vs. 59.0 years and 80.9% male in controls and HNC, respectively. Smoking, drinking (>12 std drinks/week) and most comorbidities were relatively balanced across cohorts except for respiratory and endocrine disorders present only in small number of controls and history of other cancers, which were present in HNC. More specifically, in healthy controls there were 3 subjects with diabetes (Type II-no insulin), 1 with oesophageal reflux disease, 2 with gastrointestinal disease (colitis and irritable bowel syndrome), 2 with respiratory (asthma and emphysema), 1 with hypothyroidism, while 1 subject had previously been treated for skin cancer. In HNC, 2 patients had diabetes (Type II- no insulin), 1 had oesophageal reflux disease, 1 had gastrointestinal disease (oesophagitis) while 4 patients had been previously treated for cancers (2 for prostate and 1 each for uterus and skin). No subjects in either group had obstructive sleep apnoea.

### 3.2. Comparison of Oral Microbiome Diversity between Distinct Oral sites

**Figure 1:**
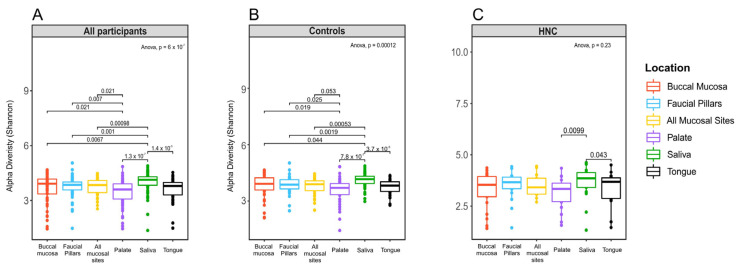
Alpha-diversity values grouped by sampling site: (**A**) samples from both healthy controls and head and neck cancer patients, (**B**) samples from healthy controls only, (**C**) oral samples from HNC only.

The analysis of microbiome alpha-diversity was performed using Shannon index values in the 27 healthy controls and 21 HNC participants to assess richness and evenness of microbiome collected from different oral sites. The results show that for all participants, microbiomes derived from different oral sites consisted of varying degrees of diversity according to individual microbiome niches in the oral cavity with median alpha values ranging from 4.10 (saliva) to 3.57 (palate) and varying significantly across the six sites (*p*  <  0.0001) (Figure 1a). When samples were stratified into controls and HNC patients, oral microbiomes from different sites in healthy controls also exhibited significant differences in microbiome diversity (Figure 1b). Oral microbiomes in HNC patients showed similar but non-significant differences among oral sites (Figure 1c). In both cohorts, saliva had the highest alpha diversity (4.16 in healthy controls, 3.85 in HNC) and palate had the lowest diversity (Figure 1b,c).

**Figure 2:**
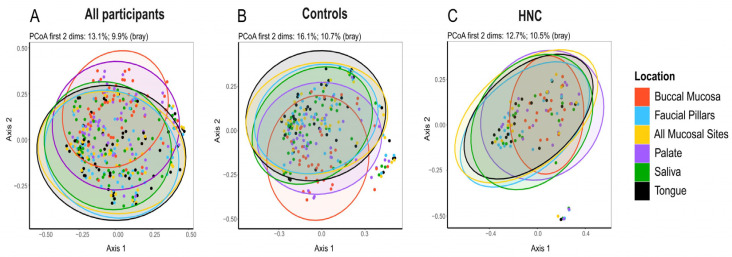
Principle Coordinate Analysis (PCoA) of the relative abundances of OTUs between locations using Bray Curtis similarity analysis: (**A**) both healthy controls and head and neck cancer patients (PCoA first 2 dimensions: 13.1%; 9.9%) (**B**) healthy controls only (PCoA first 2 dimensions: 16.1%; 10.7%), (**C**) HNC patients only (PCoA first 2 dimensions: 12.7%; 10.5%).

Similar to what was observed by alpha-diversity analysis, principal co-ordinate analysis (PCoA) of beta-diversity using the Bray Curtis dissimilarity index identified discernible patterns (*p* = 0.001) corresponding to the different oral micro-habitats, confirming significant differences among site-specific microbiomes (Figure 2a). In both HNC and healthy controls, beta-diversity showed statistically significant differences among distinct oral microbiome niches (Figure 2b; *p* = 0.001 and Figure 2c; *p* = 0.029). Pairwise comparison of beta diversity among sites found similarities between saliva, tongue and faucial pillars, while buccal mucosa and palate were similar to each other in healthy controls (Table 2). In head and neck cancer patients, pairwise comparison found no difference in microbiome composition among oral sites except for buccal mucosa, which was significantly different from all other sites (Table 2).

### 3.3. Within-Subject and between-Subject Variation

**Figure 3:**
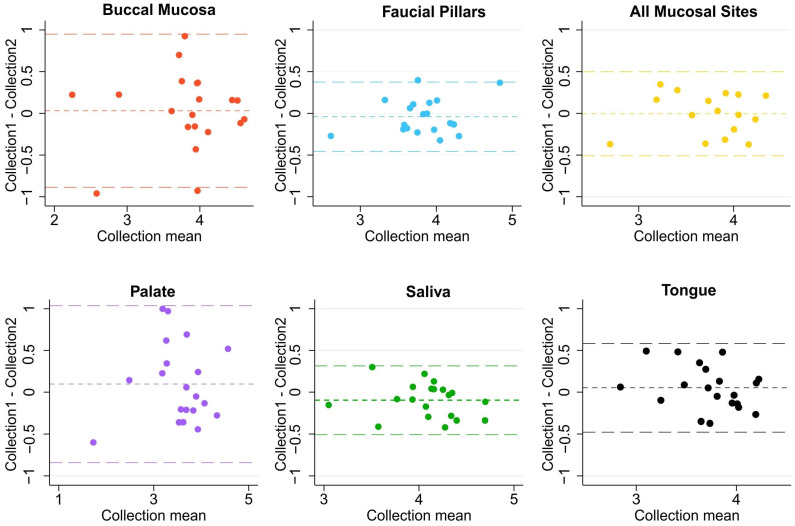
Bland-Altman plots of Shannon index of microbiome diversity collected from six different oral sites on two occasions in healthy controls.

In 20 non-HNC controls, microbiome samples from saliva, tongue buccal mucosa, faucial pillar, palate, and overall sweep of all four mucosal sites were collected at two time points, roughly a month apart. The Bland-Altman analysis of the Shannon diversity of collected microbiomes from each oral site found a mean difference of approximately zero indicating no consistent bias for all locations. Saliva and faucial pillars had the narrowest limits of agreement (+/−2 SD) with [−0.51, 0.31] and [−0.45, 0.37], respectively. This was closely followed by mucosal sweep [−0.50, 0.49] and tongue [−0.48, 0.58]. Buccal mucosa [−0.89, 0.95] and hard palate [−0.84, 1.03] demonstrated the largest temporal variability among all oral sites (Figure 3).

**Figure 4:**
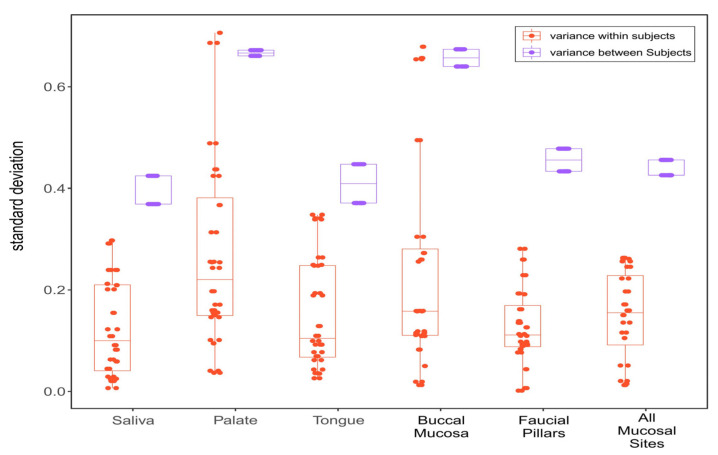
Within-subject and between-subject variance measured as standard deviation in Shannon diversity index from all six sites in healthy controls.

To assess within-subject variance, the standard deviations of each oral site’s microbiome diversity (Shannon) collected from the same individual at two different time points were compared. Similarly, standard deviations of microbiome diversity among subjects were compared to assess between-subject variance (Figure 4). As expected, microbiomes demonstrated larger between-subjects than within-subjects variation for each oral site. Saliva was found to have the least variance both within- and between subjects with (SD) 0.10 and 0.39, respectively) followed closely by tongue (0.11 and 0.41), faucial pillars (0.11 and 0.45), and mucosal sweep. Hard palate (0.22 and 0.67) and buccal mucosa (0.16 and 0.66) were found to have larger variations especially between subjects.

### 3.4. Oral Microbiome Profile of Saliva and Other Mucosal Sites

**Figure 5:**
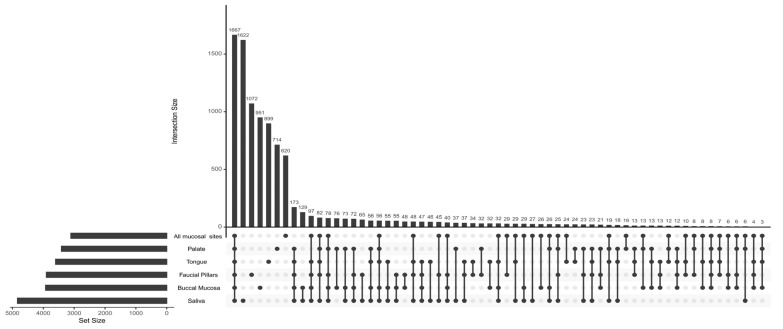
Intersections between the observed OTUs from different locations in healthy controls.

Feature intersection of operational taxonomic units (OTUs) of oral microbiomes showed that the majority of OTUs were shared among all oral sites in both healthy controls and the head and neck cancer population. (Figure 5). Overall, 1013 OTUs were shared across all sites and 1218 were shared between at least two oral sites. Saliva consisted of the most unique OTUs (551) followed by buccal mucosa (421), faucial pillars (310), tongue (289), all mucosal sites (277) and palate (275). Additionally, Saliva shared most OTUs with at least one other site (891) followed by buccal mucosa (780), faucial pillars (694), all mucosal sites (651), palate (568) and tongue (513).

**Figure 6:**
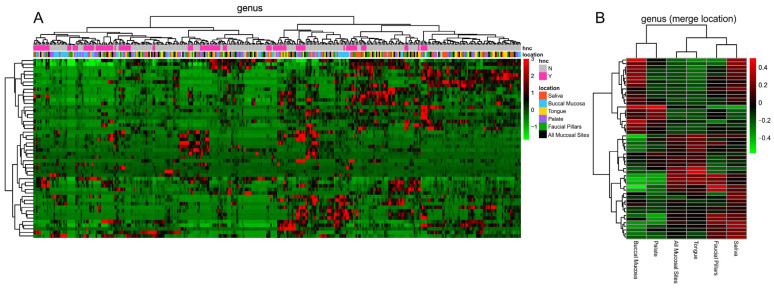
(**A**) Heatmap representation of genera detected in each sampled site from each enrolled subject. (**B**) Heatmap representation of genera when sampled sites were clustered.

Overall, oral microbiome analysis detected at least 397 bacterial genera from 25 phyla. In particular, as shown in Figure 6, the analysis showed that specimens were not recognised in clustered group, which was expected due to inter-individual variability. But the differences among oral microhabitats became evident when specimens were analysed based on specific sampled sites (Figure 6). Microbiome compositions were found to be more similar among saliva, faucial pillars, tongue, and all mucosal sites swabs whereas microbiomes derived from palate and buccal mucosa showed similarity between each-other.

**Figure 7:**
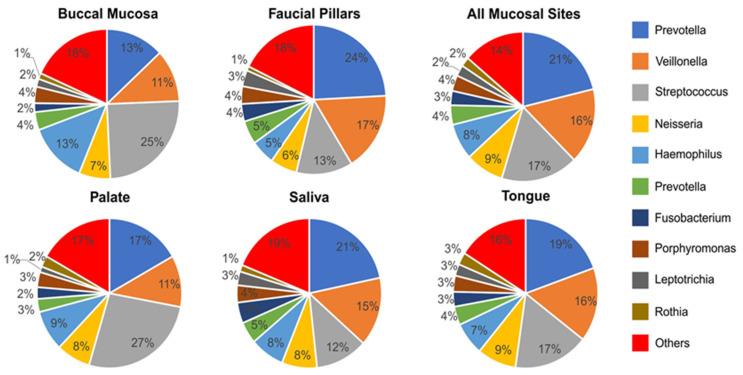
Percentage distribution of top 10 genera at each site in the oral cavity (all samples from HNC and healthy controls).

*Prevotella, Streptococcus* and *Veillonella* were the most prevalent genus each with double-digit representation making up about 50% of the total bacterial genera at each oral site (Figure 7). However, prevalence of these genera varied significantly from site to site. In particular, *Prevotella* was the most abundant and about evenly represented genus in saliva (21%), faucial pillars (24%), tongue (19%) and all mucosal sites (21%). It was the second most abundant genus in palate (17%) and buccal mucosa where it made up only 13% of total bacteria. *Streptococcus* was highly abundant and dominated palate (27%) and buccal mucosa (21%) samples, but represented a significantly lower proportion of bacteria in saliva (12%) and faucia (13%). *Veillonella* was the third most abundant genus consisting of 11% to 17% of total bacteria at each oral site. *Neisseria* was more evenly distributed among each site with prevalence ranging from 6% to 9%. *Haemophillus* consisted of 5–9% in all oral sites except in buccal mucosa where it was more abundant at 13%. *Prevotella* (*Paraprevotellaceae* family), *Fusobacterium, Porphyromonas, Leptotrichia* and *Rothia* were also highly prevalent in all sites, ranging from 1–5% of total bacteria detected. All other bacteria with relative prevalence rates less than 1% made up between 14 and 19% of total bacteria at each oral site.

### 3.5. Comparison of Oral Microbiome between Healthy Controls and HNC

**Figure 8:**
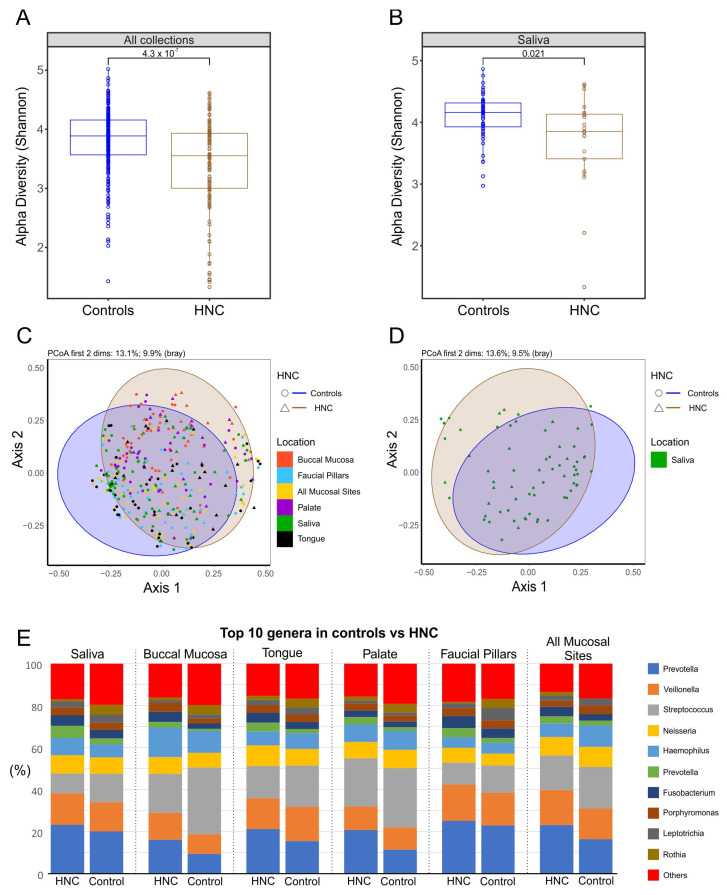
Comparison of microbiomes between healthy controls and HNC: (**A**) alpha-diversity values when all sites were combined; (**B**) Alpha-diversity values when only saliva was analysed; (**C**) principle coordinate analysis (PCoA) of the relative abundances of OTUs between location using Bray–Curtis similarity analysis when all sites were combined; (**D**) principle coordinate analysis (PCoA) of the relative abundances of OTUs between locations using Bray–Curtis similarity analysis when only saliva was analysed; (**E**) top 10 genera at each site compared between healthy control and HNC.

The microbiome alpha-diversity of 27 healthy controls and 21 HNC patients was measured using Shannon index values to assess differences in oral microbiome diversity. The results show a significant difference in alpha diversity between healthy controls and HNC patients. HNC patients’ oral microbiomes were found to have significantly lower diversity (alpha value 3.55) compared with healthy controls (alpha value 3.89) when samples from all oral sites were analysed together (*p* < 0.0001) (Figure 8). The decrease in diversity of oral microbiomes was evident when microbiomes from saliva were analysed separately (*p* = 0.021). Similarly, beta-diversity analysis showed significant differences between composition of microbiomes from healthy controls and HNC patients when all sites were analysed together (*p* = 0.01), as well as when saliva microbiomes were compared separately (*p* = 0.02).

**Figure 9:**
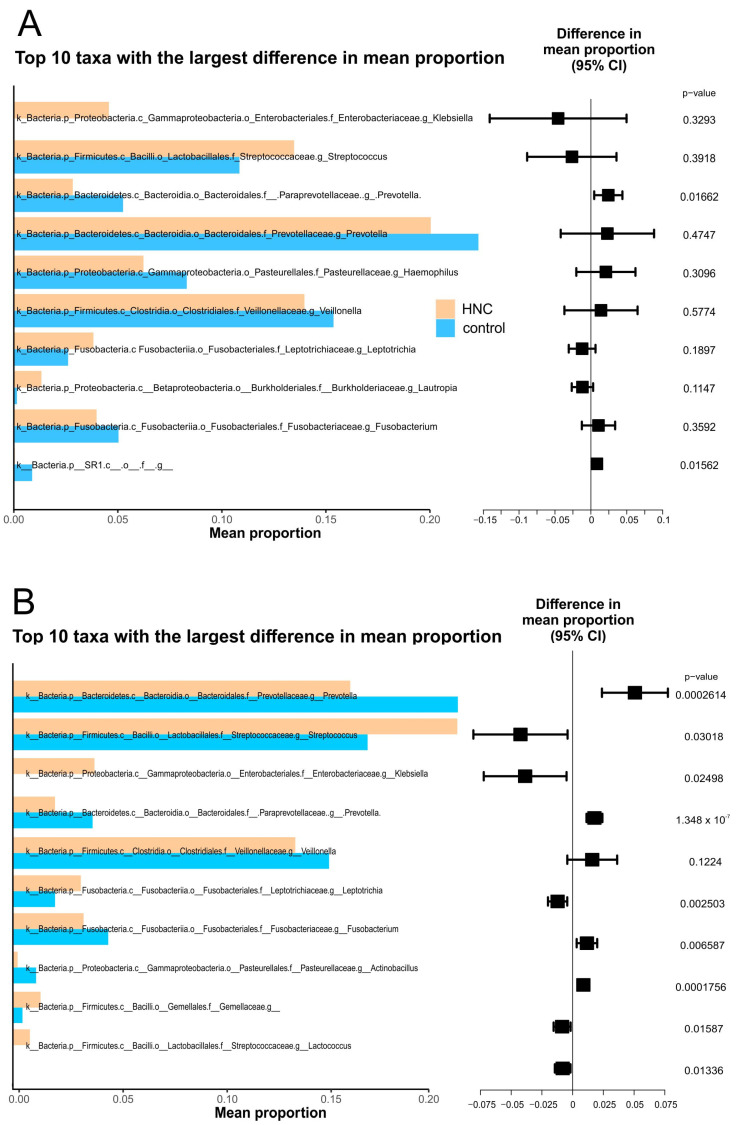
Top 10 taxa with largest difference in mean proportion. (**A**) saliva only. (**B**) all sites combined.

The top 10 genera with the highest mean relative abundance were similar between both cohorts with the exception of *Klebsiella*, which was found to be highly enriched in HNC compared with healthy controls. When all sites were analysed together, the prevalence of *Prevotella* was significantly lower in HNC patients, while *streptococcus* was highly prevalent (Figure 9A). Similarly, *Fusobacterium, Prevotella* from the *Paraprevotellaceae* family and *Veillonella* also had lower abundances in HNC patients while *Leptotrichia* tended to be highly enriched. *Neisseria, Haemophillus* and *Porphyromonas* were found to have similar abundances between both groups. Other less prevalent genera such as *Actinobacillus* were lower in abundance while *Lactobacillus* and genera from the *Gemellaceae* family were enriched in HNC cohorts (Figure 9). Similar differences in mean relative abundances of the most prevalent genera were observed in saliva from HNC and healthy controls, although most differences found in saliva were not statistically significant (Figure 9B).

## 4. Discussion

The present study aimed to characterise the diversity, stability, and composition of oral microbiomes from saliva and distinct mucosal niches in the oral cavity and establish an optimal location for oral-sample collection that can be suitable for the purpose of studying the role of oral-microbiome dysbiosis in HNC and treatment-related toxicities from chemo-radiotherapy. To evaluate microbiome diversity of each individual site, samples from four oral mucosal sites, including tongue, buccal mucosa, faucia and hard palate, all mucosal sites combined, and saliva were analysed. The results showed a significant difference in microbiome diversity and composition between individual oral sites. Saliva was found to be the most stable within-subjects (temporal) as well as between-subjects while buccal mucosa and hard palate were found to be the least stable. Saliva microbiomes had the highest diversity and were found to be more similar those of to tongue and faucia, while microbiomes from buccal mucosa and hard palate were similar to each other. Microbiome-profile analysis showed that most taxa were shared among all sites with saliva having the highest number of unique and shared bacterial species among all sites. In addition, saliva microbiomes were found to be enough for discriminating between healthy control and head HNC patients.

Comparing diversity and richness of oral microbiomes showed significant differences between individual oral-sampling sites in healthy controls. Oral microbiomes in HNC patients showed similar but non-significant differences among oral sites. Saliva microbiomes demonstrated the highest alpha diversity in both healthy controls and HNC patients as measured using the Shannon diversity index, followed in order by buccal mucosa, faucial pillars and tongue, while the microbiome from hard palate was found to be the least diverse. This result agrees with recent studies that found microbiome samples from saliva to be the most diverse, excluding dental plaques [26,33]. Beta-diversity analysis showed that the composition of microbiomes from distinct oral sites are significantly different from one another. In healthy controls, microbiomes from saliva, tongue and faucial pillars were found to be more comparable, while buccal mucosa and palate were more distinct and only similar to each other. In HNC, there were overall differences among various oral sites, but only buccal mucosa was found to be significantly different from all other sites. This is in contrast to results from Xu et al., 2015, that found buccal mucosa and salivary microbiomes to have significant overlap, although they did not include microbiomes from other mucosal sites [34]. However, the most recent study comparing saliva and buccal mucosa found that salivary microbiome composition differed from that of the buccal mucosa and showed higher richness and diversity in agreement with our study [33].

We further aimed to evaluate temporal stability of microbiomes collected from healthy controls to look at time-dependent fluctuations of samples collected from individual oral sites. The results showed saliva microbiomes to be the most stable while buccal mucosa and palate were found to fluctuate more between the two timepoints. Saliva was also found to have less inter-subject variability. This finding is important in the light of a recent study by Esberg et al., 2022, which found saliva and tooth biofilm to vary from subject to subject in small timescales of 1–3 days [35]. Their study was limited by a small number of subjects (n = 6) who were sampled daily over a 3-day period. Another study using 24 participants found saliva microbiomes collected three days apart to be stable in terms of diversity and composition [36]. Our study involving 20 healthy participants over a longer period of 4–6 weeks shows that salivary microbiomes are relatively stable in comparison to other oral sites, which can be more relevant for longitudinal clinical studies involving systemic diseases like cancer and its treatment. In contrast, buccal mucosa, despite demonstrating higher diversity, was shown to have higher temporal and inter-subject variability. Previous studies had suggested buccal mucosa as one of the potential sites for oral microbiome collection based on single-timepoint sample collection in healthy controls [34]. However, its high temporal variability makes it less desirable to use in longitudinal studies evaluating temporal changes in microbiomes due to cancer or treatment interventions like CRT.

Analysis of the observed number of operational taxonomic units, which approximate taxa present in a given sample, shows saliva not only to have the largest number of unique taxa but also to share the highest number of taxa with other oral sites. Overall, oral microbiomes consisted of about 400 bacterial genera from 25 phyla. When these genera were analysed based on their sites of origin, saliva formed a cluster with tongue and faucia, while hard palate and buccal mucosa seemed to differ based on prevalence of the most abundant bacterial genera in the oral cavity. This is consistent with our beta-diversity analysis that also found the hard palate and buccal mucosa microbiome compositions to be significantly different from those of saliva, tongue and faucia. Taken together, this result supports the findings that saliva consists of an aggregate of shedding from oral surfaces with the throat, tongue, and tonsils as the main sites of origin [37].

A more detailed analysis of prevalent genera showed that bacteria belonging to *Prevotella, Streptococcus* and *Veillonella* made up half of the total microbiome collected from each oral site. Of these, *Prevotella* was found to be more abundant on faucial pillars and saliva but were far less represented in buccal mucosa. *Streptococcus* dominated hard palate and buccal mucosa while saliva and faucial pillars consisted of lower percentages. *Veillonella* and *Neisseria* were distributed more evenly making them the third and fourth most abundant genera at each oral site. *Haemophillus* was also evenly distributed among all oral sites other than buccal mucosa. Our results are in broad agreement with results from previous studies that showed microbiome profiles from buccal mucosa and palate to be different from those of tongue, throat, and saliva [38,39,40].

The above findings suggest that saliva would be the optimal oral-microbiome sample based on higher diversity and temporal stability, as well as abundance and overlap with other sites. Next, we wanted to establish whether saliva microbiomes were enough to detect shifts based on the presence of HNC. The results show that the saliva microbiomes from healthy controls differed significantly from those of HNC. Saliva microbiomes demonstrated higher diversity in healthy controls than in HNC patients, which was also seen when samples from oral sites were analysed together. Similarly, beta-diversity analysis showed significant differences between the compositions of microbiomes from healthy controls and HNC patients’ saliva microbiomes, similar to the microbiomes from all sites combined. When the top 10 genera based on relative abundance were analysed, all sites between both cohorts had similar relative abundances with exception to Klebsiella, which was found highly enriched in HNC patients. Klebsiella spp. are opportunistic pathogens and are more prevalent in hospital environments [41]. Comparison of the top 10 taxa with the highest differences in mean proportions found saliva to reflect similar differences as from microbiomes pooled from all sites, although in saliva, differences in most taxa did not reach statistical significance. This discrepancy is most likely due to the small sample size when only one site was analysed. In future studies, we suggest using bigger sample sizees and/or metagenomics approaches for sequencing that will improve the resolution of identified taxa and give information about their functions.

Although, our results suggest saliva to have the optimal microbiome based on diversity, temporal stability, overlap with other sites and ability to detect microbiome profile changes based on presence of HNC, the study had few limitations due to logistics, time, and cost considerations. Mainly, we did not include dental plaque sampling due to logistics and the scope of this study. We excluded plaque from this study because plaque microbiomes are consistently found to be distinctive from mucosal sites and saliva and vary based on location in the oral cavity [16,26,38,42,43,44]. Therefore, collecting an adequately representative dental plaque sample at different time-points can be difficult and time consuming. Also, in this study we wanted to focus on mucosal samples and saliva due to their ease of collection as well as the fact that HNC has squamous cell origins, which makes mucosal and salivary microbiomes more relevant to the disease. We chose unstimulated saliva because microbiome diversity of unstimulated saliva has been found to be comparable to oral rinse and stimulated saliva [45]. Another obvious limitation is to not include the effects of risk factors such as smoking and drinking and other diseases as potential biases. We mitigated this limitation by including a similar number of participants with and without risk factors and known comorbidities from both cohorts. Periodontal disease can also affect the oral microbiome [46], and this has not been evaluated in this study. Lastly, we used amplicon sequencing, which can have lower resolution and is limited by the reference genomes in the database, which prevented us from further investigations involving specific species or strains of bacteria. Employing whole genome sequencing (WGS) when using saliva could enable evaluation of microbiome profile changes with greater resolution, as well as assessment of functional attributes of specific microbiota profiles.

We suggest future research focusing on microbiome dysbiosis in systemic diseases such as HNC and its impact on treatment outcomes and development of side effects such as mucositis, fibrosis, and long-term dysphagia. Specific bacteria species identified in clinical studies should be further studied in animal models to assess any potential causal relationships.

## 5. Conclusions

In conclusion, saliva microbiomes were found to be the most diverse, demonstrated higher temporal stability and being adequate for distinguishing HNC microbiomes from healthy controls making saliva an ideal sample-collection site for oral microbiome studies of progression of systemic diseases such as HNC and their role in the effectiveness and side effects of treatments.

## Figures and Tables

**Table 1:** Population and demographics.

	Non-HNC Controls (n = 27)	Head and Neck Cancer (n = 21)	*p*
Age (years) (±SD)	63.6 (±12.4)	59.0 (±14.6)	0.357
Gender(M)	70.3%	80.9%	0.849
Smokers	22.2%	38.1%	0.3788
Drinkers (>12/week)	38.1%	38.1%	0.758
Diabetes	11.1%	9.5%	0.766
Reflux	3.7%	4.7%	0.585
Gastrointestinal	7.4%	4.7%	0.822
Respiratory	7.4%	-	0.585
Cancer (previous/Non-HNC)	3.7%	19.1%	0.065
Endocrine	3.7%	-	0.884
OSA	-	-	-

**Table 2:** Distance based permutation multivariate analysis of variance (PERMANOVA) to test the null hypothesis that there were no differences in the microbial community structure across locations. * significance level of *p* < 0.05 based on 999 permutations.

Location		Controls		HNC	
		F	Bray *p*-Value *	F	Bray *p*-Value *
Buccal Mucosa	Faucial Pillars	4.41	0.001	2.86	0.001
	All Mucosal Sites	4.29	0.001	1.76	0.011
	Palate	1.45	0.093	0.54	0.975
	Saliva	3.42	0.001	1.85	0.004
	Tongue	5.35	0.001	2.62	0.001
Faucial Pillars	All Mucosal Sites	0.57	0.946	0.50	0.986
	Palate	2.84	0.001	1.62	0.034
	Saliva	0.76	0.755	0.31	0.999
	Tongue	0.86	0.604	0.65	0.879
All mucosal sites	Palate	2.34	0.001	0.91	0.589
	Saliva	0.93	0.488	0.46	0.997
	Tongue	0.19	1	0.47	0.988
Palate	Saliva	2.82	0.001	1.15	0.256
	Tongue	2.97	0.001	1.47	0.071
Saliva	Tongue	1.32	0.163	0.60	0.947

## Data Availability

The data presented in this study have been submitted to the BioProject database, reference number PRJNA901226. Public access will be available from03/11/2023 at http://www.ncbi.nlm.nih.gov/bioproject/901226.
